# Human-Computer Interaction with Detection of Speaker Emotions Using Convolution Neural Networks

**DOI:** 10.1155/2022/7463091

**Published:** 2022-03-31

**Authors:** Abeer Ali Alnuaim, Mohammed Zakariah, Aseel Alhadlaq, Chitra Shashidhar, Wesam Atef Hatamleh, Hussam Tarazi, Prashant Kumar Shukla, Rajnish Ratna

**Affiliations:** ^1^Department of Computer Science and Engineering, College of Applied Studies and Community Services, King Saud University, P.O. BOX 22459, Riyadh 11495, Saudi Arabia; ^2^College of Computer and Information Sciences, King Saud University, P.O. Box 51178, Riyadh 11543, Saudi Arabia; ^3^Department of Commerce and Management, Seshadripuram College, Seshadripuram, Bengaluru-20, India; ^4^Department of Computer Science, College of Computer and Information Sciences, King Saud University, P.O. Box 51178, Riyadh 11543, Saudi Arabia; ^5^Department of Computer Science and Informatics, School of Engineering and Computer Science, Oakland University, 318 Meadow Brook Rd, Rochester MI 48309, USA; ^6^Department of Computer Science and Engineering, Koneru Lakshmaiah Education Foundation, Vaddeswaram, Guntur 522502, Andhra Pradesh, India; ^7^Gedu College of Business Studies, Royal University of Bhutan, Gedu, Bhutan

## Abstract

Emotions play an essential role in human relationships, and many real-time applications rely on interpreting the speaker's emotion from their words. Speech emotion recognition (SER) modules aid human-computer interface (HCI) applications, but they are challenging to implement because of the lack of balanced data for training and clarity about which features are sufficient for categorization. This research discusses the impact of the classification approach, identifying the most appropriate combination of features and data augmentation on speech emotion detection accuracy. Selection of the correct combination of handcrafted features with the classifier plays an integral part in reducing computation complexity. The suggested classification model, a 1D convolutional neural network (1D CNN), outperforms traditional machine learning approaches in classification. Unlike most earlier studies, which examined emotions primarily through a single language lens, our analysis looks at numerous language data sets. With the most discriminating features and data augmentation, our technique achieves 97.09%, 96.44%, and 83.33% accuracy for the BAVED, ANAD, and SAVEE data sets, respectively.

## 1. Introduction

Speech emotion recognition (SER) is a new study area in human-computer interaction. Emotional understanding is critical in human social relationships. Despite being researched since the 1950s, the study of emotional signals has made significant breakthroughs in recent years [1,2]. Because emotion identification via face recognition is technically hard, real-time implementation is prohibitively expensive. Because high-quality cameras are required for obtaining facial photographs, the cost of implementation is likewise considerable. Aside from human facial expressions, language is a more appropriate channel for expression identification. Vocal emotions are crucial in multimodal human-computer contact [3,4]. Language emotion acknowledgment, in general, is a critical subject because speech is the primary medium of human communication. SER has progressed from a minor concern to a serious one in human-computer contact and speech processing in the recent decade. SER offers a broad range of possible uses. Human-computer interfaces, for example, might be programmed to behave differently depending on the user's emotional state. This may be particularly critical when voice is the major contact form with the machine [5]. Language has two sorts of information: textual information and emotional information. The machine can accomplish automated emotional identification of voice signals to create a harmonious human-computer connection experience. Voice may be used to assess a client's emotions in a customer service system. It may boost children's social-emotional abilities and academic skills in the educational assistance system [6]. Problems may be dealt with by parents and teachers promptly.

The study of feelings in human-computer contact is a burgeoning field of study. Emotions and human behavior are inextricably linked. Moreover, computer emotion identification may provide humans with a satisfying human-computer connection interface. Speech-based emotion identification has been extensively employed in human-computer contact due to new applications in human-machine connections, human-robot interfaces, and multimedia indexing. Scientific improvements in capturing, storage, and processing audio and visual material; the growth of nonintrusive sensors; the introduction of wearable computers; and the desire to enhance human-computer interaction beyond point-and-click to sense-and-feel are all causes for fresh concern.

Affective computing, a discipline that develops devices for detecting and responding to user's emotions [7], is a growing research area [8] in human-computer interaction. It is a science that creates systems for recognizing and reacting to human emotions (HCI). The primary goal of affective computing is to gather and analyze dynamic information to improve and naturalize human-computer interactions. Affective mediation, a subset of affective computing, employs a computer-based system as a mediator in human-to-human communication, expressing the emotions of the interlocutors [7]. Emotive mediation attempts to reduce the filtering of affective knowledge by communication systems, which are often committed to the spread of verbal material and ignore nonverbal material [9]. Other uses of this form of mediated communication exist, such as textual telecommunication (effective electronic mail, affective chats). Speech emotion recognition (SER) is another hotly debated area of HCI research [10]. Concerning this issue, Ramakrishnan and El Emary [11] presented different applications to demonstrate the relevance of SER approaches.

Feelings are physiological stages of varied sensations, thoughts, and behaviors of connected individuals and psychological and physiological responses to numerous external stimuli. Feelings have a vital role in both everyday life and work. In several disciplines, it is critical to detect emotions accurately. Emotion recognition research has been used in psychology, emotional calculation, artificial intelligence, computer vision, and medical therapy, among other fields [12–14]. Emotion identification, for example, may aid in the identification of depression, schizophrenia, and different mental illnesses. It may help physicians grasp their patients' genuine feelings. Moreover, computer emotion identification may provide humans with a satisfying human-computer connection interface.

Different techniques have been developed to find the emotions by researchers, such as computer vision, neural networks, machine learning, and signal processing. The proposed emotion recognition system was with a combination of multiple handcrafted features. In order to improve the identification rate, we combined all the methods in one input vector. Thus, we chose to use the coefficients MFCC, Chroma, and ZCR in our study because these methods are more used in speech recognition, and they receive good recognition rates. The classification task was performed on multiple traditional machine learning classifiers along with the designed 1D CNN.

### 1.1. List of Contributions

  A study of the emotion classification on Arabic language speech, which is a less studied area.
  A customised CNN model for identifying and classifying the emotion from the speech signals. The model was primarily developed Basic Arabic Vocal Emotions Dataset (BAVED) data set [15], which consists of emotions classified into three classes: low, normal, and high.
  Through the input speech emotion signals, features were extracted with the help of the feature extraction technique. Various kinds of feature extraction techniques were included in the proposed methodology. A study of different combinations of features on classification performance is also presented.
  Data augmentation to address challenges of class imbalance, data scarcity, and hence performance improvement.
  Study of other language databases with complex emotions. Experiment results show the validity of our proposed method on other SER tasks with more complex emotions.

The remainder of this article is organised as follows.  Section 2 summarises earlier research in the same field of study.  Section 3 explains the experimental procedure and the details of parameter setting. The outcomes of the experiment are analysed and described in Section 4. Conclusions are provided in section 5, followed by the references.

### 1.2. Literature Review

Numerous articles have been published that demonstrate how to detect emotions in speech using machine learning and deep learning techniques. For researchers, selecting strong traits for SER is a challenging task. Several researchers have benefited from the unique properties of SER. The mainstream of low-level prosodic and spectral auditory properties, including fundamental frequency, formant frequency, jitter, shimmer, speech spectral energy, and speech rate, have been linked to emotional intensity and emotional processes [16–18]. Complex parameters, like Mel-frequency cepstral coefficients (MFCCs), spectral roll-off, Teager Energy Operator (TEO) characteristics [19–21], spectrograms [22], and glottal waveform characteristics, all produced favorable SER results [23–25]. For instance, Dave [26] evaluated a variety of features for speech emotions. They demonstrated the superiority of preferable Mel frequency cepstral coefficient (MFCC) [27] features for SER over other low-level features, such as loudness, linear productivity code (LPC) [28], and so on. According to Liu [29], compared with MFCCs that include additional speech features such as jitter and shimmer, gamma-frequency cepstral coefficient (GFCC) characteristics for SER may enhance unweighted accuracy by up to 3.6%. Liu et al. [30] proposed an approach for SER that makes use of a Chinese speech data set [31] (CASIA) to choose hidden emotional features based on correlation and a decision tree based on an extreme learning machine (ELM) for classification. Fahad et al. [32] devised an approach for choosing glottal and MFCC characteristics for training DNN-based models for SER.

Noroozi et al. [33] proposed a method for identifying adaptable emotions based on visual and acoustic data processing. In his research, they retrieved 88 features (Mel frequency cepstral coefficients (MFCC), filter bank energies (FBEs)) using Principal Component Analysis (PCA) to decrease the measurement of earlier extracted features. Bandela and Kumar [34] detected five emotions using the Berlin Emotional Speech database by combining an acoustic characteristic known as the MFCC with a prosodic property known as the Teager Energy Operator (TEO) (2017). Zamil et al. [35] classified the seven emotions using the Logistic Model Tree (LMT) technique with a 70% accuracy rate, utilizing the 13 MFCC gathered from auditory figures in their recommended method. All of this work emphasizes some aspects while neglecting others. Additionally, when such approaches are used, accuracy cannot exceed 70%, which may affect the capacity to perceive emotion in speech. According to several authors, the most critical audio aspects for emotion detection are the spectral energy distribution, the Teager Energy Operator (TEO) [36], the MFCC, the MFCC, the Zero Crossing Rate (ZCR), and the filter bank energies (FBE) energy parameters [37]. On the other hand, Kacur et al. [38] attempted to explain how, in addition to speech signal features, common processing procedures, such as segmentation, windowing, and preemphasis, have an impact on the model's performance.

Numerous research articles examined the use of convolutional neural networks (CNNs) to detect whole language spectrogram arrays or isolated bands of spectrograms to determine speech emotions [39,40]. Fayek et al. [41] used a DNN to extract SER from small settings of communication spectrograms. The average accuracy was 60.53% (when using the eNTERFACE database) and 59.7% (when using the SAVEE database). A similar but superior method produced an average accuracy of 64.78% (IEMOCAP data with five classifications) [42]. Several chain structures comprising CNNs and recurrent neural networks (RNNs) were trained on EMO-DB data using communication spectrograms [43]. The most acceptable arrangement produced a usual accuracy of 88.01% and a recall of 86.86% for seven emotions. Han et al. [44] employed a CNN to extract affect-salient properties, which then were used by a bidirectional recurrent neural network to detect four emotions using IEMOCAP data. Trigeorgis et al. [45] created a CNN and LSTM-based method for spontaneous SER that uses the REmote COLlaborative and Affective RECOLA natural emotion database. Zhao et al. [46] also used a recurrent neural network (RNN) to extract relationships from 3D spectrograms across timesteps and frequencies. Lee et al. [47] developed a parallel fusion model Fusion-ConvBERT”, consisting of bidirectional encoder representations from transformers and convolutional neural networks. A deep convolution neural network (DCNN) and Bidirectional Long Short-Term Memory with Attention (BLSTMwA) model (DCNN-BLSTMwA) is developed by [48], which can be used as a pretrained for further emotion recognition tasks.

## 2. Materials and Methods

### 2.1. Data Set

The Basic Arabic Vocal Emotions Dataset (BAVED) data set [15] was used in the study. It is a collection of recorded Arabic words (.wav) in diverse emotional expressions. The seven words were indicated in integer format (0-like, 1-unlike, 2-this, 3-file, 4-good, 5-neutral, and 6-bad). The data set contains each word pronounced at three levels, each of which corresponds to a person's feelings: 0 for low emotion (tired or exhausted), 1 for neutral emotion, and 2 for high emotion positive or negative emotions (happiness, joy, sadness, anger). Each file name in the data set has six sections, which include the following information.

1. Speaker_id (int).
2. Gender of the speaker (m or f).
3. Speaker age(int).
4. Word spoken (int from 0 to 6).
5. Emotion spoken (int from 0 to 2).
6. Record id(int).

There are 1935 recordings in the data set, recorded by 61 speakers (45 men and 16 women).  Table 1 shows the distribution of voice samples among different categories present in the data set.

### 2.2. Exploratory Data Analysis (EDA)

**Figure 1:**
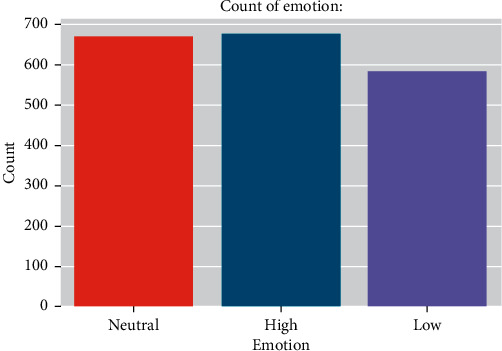
Distribution of target classes in the data set.

**Figure 2:**
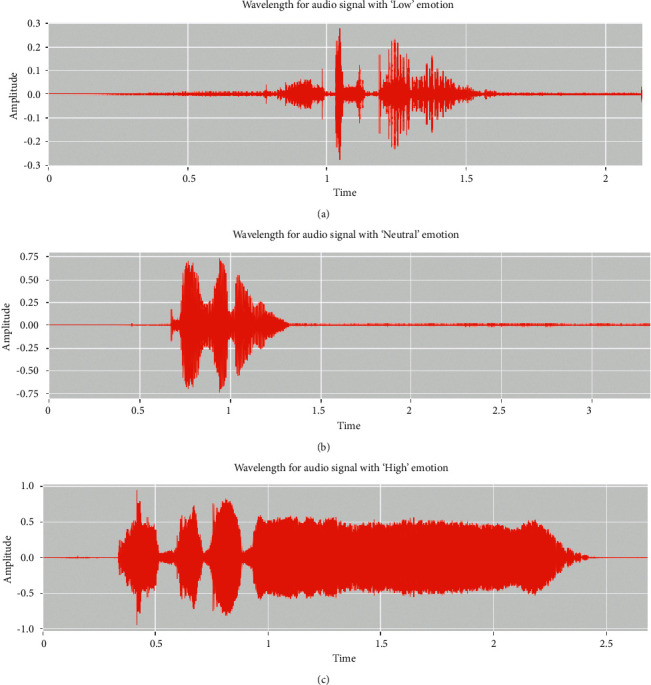
Waveforms for the three classes of emotions.

**Figure 3:**
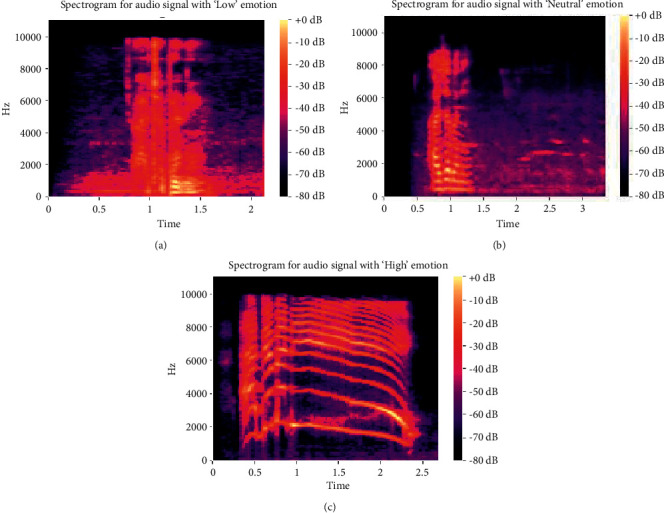
Spectrograms for the three classes of emotions.

Figure 1 depicts the distribution of emotions in the data set. The data set is slightly skewed because the number of samples in the “low” class of the database is lower than that in other classes. This could have an impact on Deep CNN's training performance. Figures 2 and 3 also showed the waveform and spectrogram for the sample voices in the data set. There is enough information in the waveform and spectrogram to distinguish the classes. Also, experimentally we aim to the conclusion that in the data set first 0.3s contains no information about emotion, and most of them are less than 2.5s.

**Figure 4:**
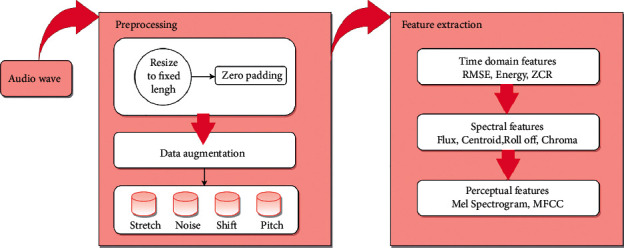
Preprocessing and feature extraction.

Before developing the model, the audio signals are subjected to preprocessing and feature extraction operations, as depicted in Figure 4. Resize to fixed length and augmentation are the processes that make up the preprocessing phase of the process flow diagram. Then, after reading the audio files in.wav format, we resize all the audio samples to be the same length by either extending their duration by padding them with silence (zeroes) or truncating their duration.

### 2.3. Data Augmentation

To address the data imbalance between emotion classes, we used a variety of strategies to increase the amount of samples in the data set.

#### 2.3.1. Noise Injection

The audio data had random noise added to it. The rate of noise to be added to the audio was set to 0.035.

#### 2.3.2. Time Shifting

It just changes the audio to the left or right for a random second. If you fast forward audio by *x* seconds, the first *x* seconds will be marked as 0. If we move the audio to the right (backward) for *x* seconds, the last *x* seconds will remain 0. We gave a random value for shifting in the range (-5 to 5) so that it will produce left and right shifts randomly on the data set.

#### 2.3.3. Time Stretching

This approach extends the time series at a constant rate. The specified rate was 0.8.

#### 2.3.4. Pitching

The audio wave's pitch is adjusted according to the provided pitch factor. The pitch factor was set to 0.7.

### 2.4. Feature Extraction

Modern deep learning on audio class recognition includes feature extraction as a key component. There are numerous ways to accomplish this. We are focusing mainly on three types of features of audio signals (Figure 4).

1. Time-domain features
2. Spectral features
3. Perceptual features

### 2.5. Time-Domain Features

#### 2.5.1. Zero-Crossing Rate

The number of zero crossings in a specific region of the signal divided by the number of samples in that region is the zero crossing rate (ZCR) [49], that is, the rate at which the signal crosses the zeroth line; more precisely, the rate at which the signal changes from positive to negative or vice versa. Mathematically, it can be measured as follows:

(1) $$\begin{array}{c} \text{ZCR}=\frac{1}{N-1}\sum_{n=1}^{N-1}\text{sign}\left(s\left(n\right)s\left(n-1\right)\right), \end{array}$$

where *s* = signal, *N* = length of a signal, and the sign(*s*(*n*)*s*(*n* − 1)) is calculated as

(2) $$\begin{array}{c} \text{sign}\left(s\left(n\right)s\left(n-1\right)\right)=\left\{\begin{array}{c} 1,\text{if }s\left(n\right)s\left(n-1\right)\geq 0\\ 0,\text{if }s\left(n\right)s\left(n-1\right)<0 \end{array}\right). \end{array}$$

#### 2.5.2. Energy

The overall magnitude of a signal, i.e., how loud it is, is the signal's energy. It is defined as in

(3) $$\begin{array}{c} E\left(x\right)=\underset{n}{\sum }{\left|x\left(n\right)\right|}^{2}. \end{array}$$

#### 2.5.3. Root-Mean-Square Energy (RMSE)

It is based on the total number of samples in a frame. It serves as a loudness indication because the more energy, the louder the sound. It is less susceptible to outliers. The square root of the mean squared amplitude over a time interval is the RMS Energy (RMSE). It is characterized by

(4) $$\begin{array}{c} {\text{RMS}}_{t}=\sqrt{\frac{1}{K}\;\sum_{k=t.K}^{\left(t+1\right)\cdot \left(K-1\right)}s{\left(k\right)}^{2}}. \end{array}$$

### 2.6. Spectral Features

#### 2.6.1. Spectral Centroid

A spectral centroid is a measurement of a sound's “brightness,” signifying the location of the center of mass of the spectrum. The spectral centroid is equivalent to a weighted median. The Fourier transform of the signals with weights can be used to determine it mathematically as in

(5) $$\begin{array}{c} \text{Centroid}=\frac{\sum_{n=0}^{N-1}f\left(n\right)x\left(n\right)}{\sum_{n=0}^{N-1}x\left(n\right)}, \end{array}$$

where *X*(*n*) is the weight frequency value. *N* is the bin number. *F*(*n*) is the center frequency of the bin.

#### 2.6.2. Spectral Flux

Spectral flux is calculated as the squared difference between the normalized magnitudes of the spectra of two consecutive short-term windows and measures the spectral change between two frames ((6)

(6) $$\begin{array}{c} {\text{Fl}}_{\left(i,i-1\right)}=\overset{{\text{Wf}}_{L}}{\underset{k=1}{\sum }}{\left({\text{EN}}_{i}\left(k\right)-{\text{EN}}_{i-1}\left(k\right)\right)}^{2}, \end{array}$$

where EN_*i*_(*k*) is the *k*^th^ normalized DFT at the *i* ^th^ frame as in

(7) $$\begin{array}{c} i.e,\;{\text{EN}}_{i}\left(k\right)=\frac{X_{i}\left(k\right)}{\sum_{l=1}^{{\text{Wf}}_{L}}X_{i}\left(l\right)}. \end{array}$$

Spectral Rolloff: It is the fraction of bins in the power spectrum below which 85% of the spectral distribution is concentrated.

Chroma**:** Chroma is a measure of each chromatic pitch class (C, C♯, *D*, D♯, *E*, F, F♯, *G*, G♯, A, A♯, B) in the audio signal. It is one of the most important aspects of audio processing.

### 2.7. Perceptual Features

#### 2.7.1. Melspectrogram

Melspectrogram is a representation of frequencies in the Mel scale. The Mel scale comprises pitches that are equally spaced for the listener. The Mel scale is based on how the human ear works, which better detects differences at lower frequencies than higher frequencies. The Fourier transform can be used to convert frequencies to the Mel scale. The major three steps for creating Melspectrogram are.

1. Compute the fast Fourier transform (FFT)
2. Generate Mel scale
3. Generate spectrogram

### 2.8. Mel-Frequency Cepstral Coefficients (MFCCs)

The envelope of the voice signal's time power spectrum depicts the vocal tract, and MFCC accurately represents this envelope. The Mel frequency cepstral (MFC) represents the short-term power spectrum of any sound, and the MFC is made up of MFCC. The inverse Fourier transform (cepstral) representation can be used to derive it. MFC allows for a better depiction of sound because the frequency bands on the Mel scale are evenly distributed in MFC, which closely approximates the human auditory system's reaction.

The total amount of extracted parameters were

- 40 Mel-frequency cepstral coefficients (MFCC)
- 128 Mel spectrogram
- 12 chromagram
- Other 6 features (RMS energy, energy, zero crossing rate (ZCR), spectral centroid, spectral flux, and spectral rolloff)

### 2.9. Model Architecture

We constructed an emotion recognition model after augmenting and preprocessing the data. To construct an emotion categorization model, various classifiers from the machine learning family have been presented. K-Nearest Neighbors, Decision Trees, Random Forest, SVC RBF, SVC, Ada Boost, Quadratic Discriminant Analysis, and Gaussian NB were among the techniques used. Hyperparameters: KNN (*K* = 3), SVC (*C* = 0.025), Decision Tree (max depth = 5), and Random forest (max depth = 5, n_estimators = 10, max features = 1).

**Figure 5:**
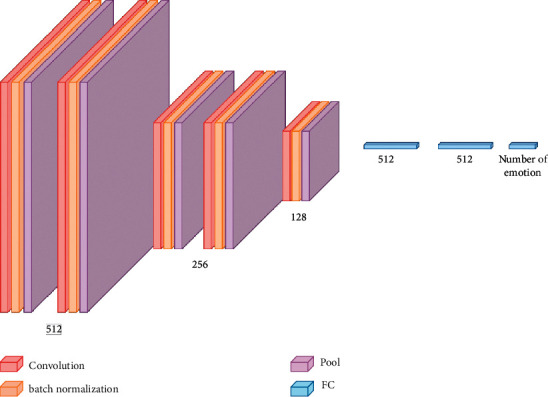
1D CNN architecture.

**Figure 6:**
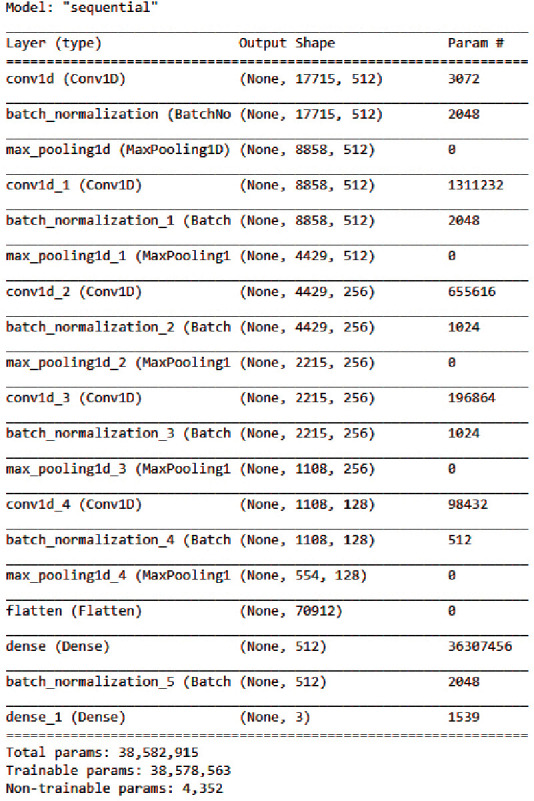
A detailed description of the 1D CNN.

This article aimed to create a 1D convolution neural network (CNN) (inspired from Aytar et al. [50] that could learn from extracted features and categorize audio signals based on emotions). However, the goal was to create an architecture with fewer parameters, which would lessen the requirement for a large data set and the computational bottleneck during training. As a result, the planned architecture (Figure 5) only had five convolutional layers interconnected by max-pooling layers. The fifth pooling layer's output is flattened and connected to fully connected (FC) layers. Overfitting was reduced by Batch normalization [51]. Three neurons at the final fully connected layer categorize objects into three classes. The baseline model takes an array of 17,715 dimensions as input, which represents the extracted features from the data set (Figure 6). To adapt the model for different applications and variable data sets, necessary changes should be made to the model architecture based on the characteristics of the input audio data to study. Depending on audio lengths and sampling rate, the number of input features may vary. The number of neurons present at the last FC layer can also be modified based on the number of target classes in the data set.

### 2.10. Training Pipeline

Test data set accounts for 20% of the data, whereas validation accounts for 10% of the remaining data. The Keras framework is used to build the full 1D CNN architecture, which is supported by TensorFlow and coded in *Python*. All other processing and analysis were done with NumPy, OpenCV, Scikit-learn, and other open-source tools. A 32 GB NVIDIA Quadro P1000 GPU was used for the training. The training began with a learning rate of 0.001 and was subsequently reduced by a factor of 0.5 after observing the validation loss. As an optimizer, we used the Adam algorithm [52]. With a batch size of 64, the training could last up to 50 epochs. However, early stopping will occur if the validation loss does not decrease continuously for a long period. The trained model is applied to the test data set to validate the model's performance.

### 2.11. Performance Evaluation Matrices

#### 2.11.1. Accuracy

Accuracy refers to the percentage of correct predictions made by our model. In classification problems, accuracy refers to the number of correct predictions made by the model across all types of predictions. The following equations show the formal definition of accuracy:

(8) $$\begin{array}{c} \text{accuracy}=\frac{\text{number of correct predictions}}{\text{total number of predictions}}, \end{array}$$

or

(9) $$\begin{array}{c} \text{accuracy}=\frac{\text{TP}+\text{TN}}{\text{TP}+\text{TN}+\text{FP}+\text{FN}}\text{.} \end{array}$$

*TP* = true positives, *TN* = true negatives, *FP* = false positives, and *FN* = false negatives.

  True positives (TP): true positives are the cases when the actual class and predicted class of a datapoint is same (both are positive)
  True negatives (TN): true negatives are the cases when the actual class and predicted class is same (both are negative)
  False positives (FP): false positives are the cases when a data point was mispredicted to belong to a class
  False negatives (FN): false negatives are the cases when a data point was mispredicted to not belong to a class

### 2.12. Recall

Recall is the proportion of actual positives predicted correctly as

(10) $$\begin{array}{c} \text{recall}=\frac{\text{TP}}{\text{TP}+\text{FN}}\text{.} \end{array}$$

### 2.13. Precision

Precision is the proportion of positive predictions that are actually correct as shown in

(11) $$\begin{array}{c} \text{precision}=\frac{\text{TP}}{\text{TP}+\text{FP}}\text{.} \end{array}$$

### 2.14. F1 Score

To completely assess a model's effectiveness, you must look at both precision and recall. Regrettably, precision and recollection are sometimes at odds. Conversely, increasing precision usually decreases recall and vice versa. The F1 score was created to solve this issue. The harmonic mean of precision and recall is the F1 score is calculated as

(12) $$\begin{array}{c} F1\text{ score}=\frac{2*\text{precision}*\text{recall}}{\text{precision}+\text{recall}}\text{.} \end{array}$$

## 3. Results and Discussion

It is required to recognize the speaker's emotions for multiple fields, including medicine, business, and criminal detection. In contrast, it is the most challenging problem as age, gender, cultural differences, and other factors influence the clarity of emotions in a person's voice. Even humans struggle to recognize the intense emotions of speech regardless of the semantic content; therefore, the capacity to do so automatically utilizing programmable devices is still a research problem.

Even though Arabic is one of the top ten most widely spoken languages, it lacks emotion and sentiment corpora [53]. This could lead to the research focusing mostly on the Arabic language. The BAVED data set's developers have stated that it should perform well in voice emotion recognition for research purposes. We also considered that developing an emotion recognition model on this data set would be beneficial because the data set comprises seven words pronounced with three different levels of emotion. On the other hand, the recognition findings cannot be taken as proof that the acted speech is similar to natural speech [54]. Designing algorithms that perform well on acted speech may be beneficial for providing a practical basis for a theory, according to Hammami [55], yet there are grounds to suspect that acted speech is different from natural speech. As a result, we attempted to create a model for another Arabic Emotion database, ANAD [56], which is entirely natural speech.

This section reports on and discusses the experimental results assessing the performance of our 1D CNN systems for speech emotion recognition on the three open-source data sets.

**Experiment 1 .**

Performance of different classification models with different combinations of features without any prior audio augmentation

The experiment aimed to clarify the significance of chosen groups of features and the classification ability of selected classification methods for speech emotion recognition systems.

### 3.1. Input Samples

BAVED data set with 3 classes of emotions, low, medium, and high.

*Feature Extraction*: computing of input vectors (speech parameters):

1. Chroma, Melspectrogram, and MFCC
2. Chroma, Melspectrogram, MFCC, Contrast, Tonnetz, ZCR, RSME, Energy, Flux, Centroid, Rolloff

### 3.2. Emotion Classification

1. 1D CNN-(Ours)
2. Other machine learning models: KNN, Random forest, SVC RBF Kernel, SVC, Decision Tree, AdaBoost, Quadratic Discriminant Analysis, and Gaussian NB

Table 2 summarises the recognition rate found for the different classification models as a function of different combinations of features. The results show that the 1D convolution gives the best performance compared with the linear and polynomial kernels.

This research discusses the impact of classification method, identifying the best combination of features, and data augmentation on speech emotion recognition accuracy. There is an increase in the system performance in terms of accuracy and system complexity by selecting the appropriate parameters in conjunction with the classifier compared with the raw waveform efforts. This phase is required, particularly for systems that are used in real-time applications. Some raw waveform efforts [57,58] that forgo hand-designed features should take advantage of the deep learning model's superior modeling power, learning representations optimized for a task [59]. This, however, raises computational costs and data requirements, and the benefits may be difficult to realize in practice. Mel frequency cepstral coefficients (MFCCs) have been the primary acoustic feature representation for audio analysis tasks for decades [60]. The first experiment in this work was to create an acceptable feature representation for this task, and we discovered that a combination of time, spectral, and perceptual features generated the best accuracy in all models we developed (Table 2).

**Experiment 2 .**

Effect of the data augmentation on a different combination of features and models

The goal of the experiment was to demonstrate the impact of data augmentation on model classification performance. On the enhanced audio data set, Experiment 1 is repeated.

Four audio augmentations were used with the audio emotion data set: noise injection, time-shifting, time-stretching, and pitching.  Table 3 also shows how different models perform when using a combination of feature extractors. It is also evident that 1D CNN designed by us outperforms the traditional machine learning classifiers.

Experiment 2 aims to determine the impact of data augmentation on the model's performance by solving the limited training data problem.  Table 3 shows how a controlled, steady increase in the complexity of the generated data makes machine learning algorithms easier to understand, debug, and improve [61,62].

**Experiment 3 .**

Performance of the designed 1D CNN model on BAVED data set

**Figure 7:**
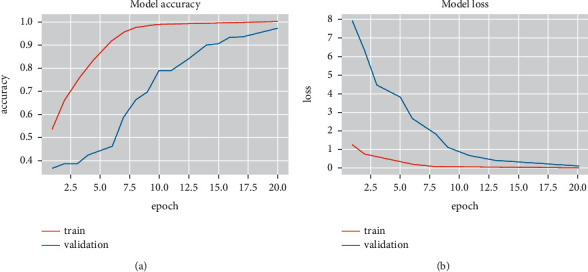
Accuracy and loss graphs of the 1D CNN.

**Figure 8:**
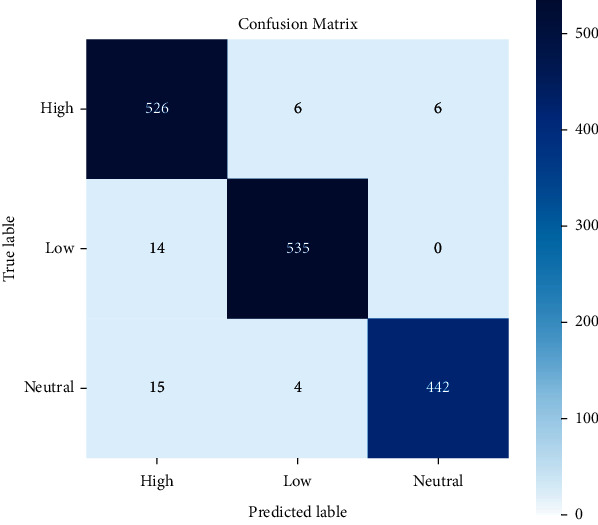
Confusion matrix for 1D model for BAVED database.

In the segment, the presentation of the proposed technique is analysed for emotion recognition using the CNN network. The investigation considered three different types of emotions: low, normal, and high. The Arabic Emotion data set BAVED was used as the basis for the research. The suggested speech recognition model is tested on features such as Chroma, Melspectrogram, MFCC, Contrast, Tonnetz, ZCR, RSME, Energy, Flux Centroid, and Rolloff from an augmented data set.  Figure 7 depicts the 1D CNN's accuracy and loss graphs. The plots show that nearer to the 20th epoch, there are evident converges. The confusion matrix (Figure 8) of the data is used in this study to examine the recognition accuracy of the distinct emotional classes. When using the BAVED data set, the 1D CNN classifier recognizes “low“ and “high“ emotions with more accuracy than the “neutral“ class (Table 4).

**Experiment 4 .**

Performance on other data sets

### 3.3. ANAD Data Set

**Figure 9:**
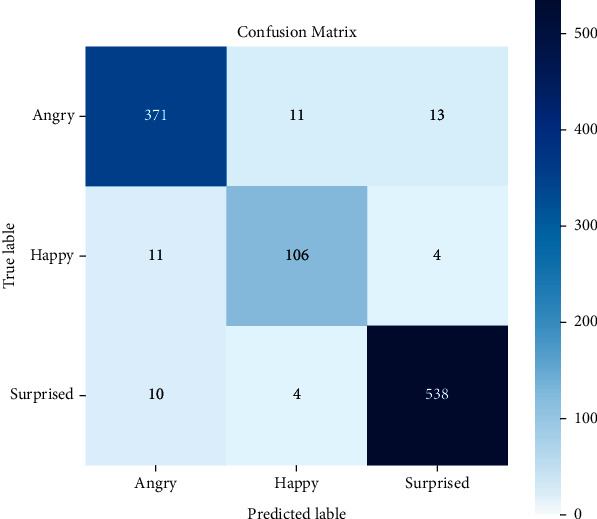
Confusion matrix of ANAD using 1D CNN.

The Arabic Natural Audio Dataset (ANAD) [56] is available online in Kaggle for emotion recognition. The audio recordings of three emotions are included in the data set: happy, angry, and surprised. The CNN classifier developed on the data set achieved an accuracy of 96.44%, with “surprised” and “angry” emotions were detected with better accuracies, as demonstrated in Table 5 and Figure 9.

### 3.4. SAVEE Data Set

**Figure 10:**
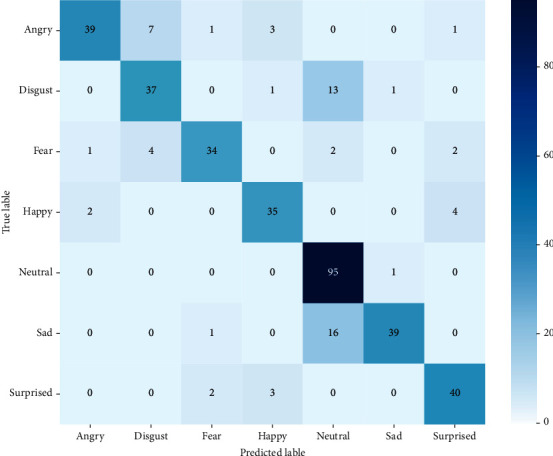
Confusion matrix of SAVEE using 1D CNN.

The SAVEE data set contains emotional utterances in British English captured from four male actors. Anger, fear, happiness, disgust, neutral, surprise, and sadness are the seven emotional states. With the SAVEE database, the 1D CNN obtained an accuracy of 83.33%.  Figure 10 depicts the confusion matrix. The emotion 'neutral' is recognized with the greatest accuracy (Table 6).

The results in Table 7 describe classification accuracy for a particular type of classifier (1D CNN) that has been trained by best-scored MFCC features of the augmented emotion data set. The classifier was trained by pair of emotions and values in the tables show tested ability to recognize emotional state.

The ability of the entire network model to distinguish emotions from audio data improves dramatically when the 1D CNN model is used instead of typical ML models in this study. Based on extracted features, the suggested method may achieve a high level of recognition accuracy. Our suggested method is highly comparable with state-of-the-art methods on the BAVED, ANAD, and SAVEE databases, according to the results in Tables 8–10. This shows how our proposed method outperforms earlier known methods.

The number of samples we collected limits the model we suggested in this study; hence, this method can only classify a restricted number of emotions with greater accuracy. The data sets we used to develop the model contained more “acted” speech than “natural” speech; it has not been employed in real-life scenarios. Furthermore, the data set is not age or gender agnostic. We can improve the algorithms with more accurate and varied data sets [67] in the future to be used in everyday life by the broader public.

## 4. Conclusions

With the advancement of ER technology, SER research is becoming more prevalent. This study looked at how to reliably discern emotion status in speech. We also discovered how data augmentation improves the model's performance. Emotions are primarily classified using SER technology by learning low-level or spectral information. The proposed approach uses CNN to classify emotions based on feature space for low-level data such as pitch and energy, and spectral features such as a log-Mel spectrogram, STFT, to learn high-level spectral properties to identify emotions. The research proposed an improved model for recognizing emotions in Arabic speech, BAVED, as pronounced by people of various ages and languages. To recognize emotions, the study also looked at the cross-corpus SER problem in two separate speech data sets, ANAD and SAVEE. According to current research, we yielded ER accuracy results of 97.09% (BAVED), 96.44% (ANAD), and 83.33% (SAVEE), respectively. This contribution is independent of language and could be used by other researchers to improve their results. Adding more speech units to the corpus would substantially aid in developing an effective classification model for recognizing distinct emotions from speech.

## Figures and Tables

**Table 1:** Data set distribution.

Emotion category		Low	Neutral	High
Gender	Male	342	379	388
	Female	243	293	290
Total number of samples		585	672	678
Total: 1935				

**Table 2:** Performance of different combinations of features and models without augmentation.

Data set	Features	Augmentation	Model	Accuracy (%)
BAVED	Chroma, melspectrogram, MFCC	No	1D convolution	81.395
			KNeighborsClassifier	79.07
			RandomForestClassifier	78.04
			SVC RBF kernel	76.74
			SVC	72.61
			DecisionTreeClassifier	68.22
			AdaBoostClassifier	66.15
			QuadraticDiscriminantAnalysis	55.30
			GaussianNB	51.68
	Chroma, Melspectrogram, MFCC, Contrast, Tonnetz, ZCR, RSME, energy, flux, centroid, rolloff	No	1D convolution	82.07
			KNeighborsClassifier	79.59
			RandomForestClassifier	79.07
			SVC RBF kernel	75.71
			SVC	73.64
			DecisionTreeClassifier	63.31
			AdaBoostClassifier	67.96
			QuadraticDiscriminantAnalysis	59.69
			GaussianNB	51.16

**Table 3:** Performance of different combinations of features and models with augmentation.

Data set	Features	Augmentation	Model	Accuracy (%)
BAVED	Chroma + Melspectrogram + MFCC	Yes	1D convolution (CNN)	96.38
			RandomForestClassifier	89.02
			KNeighborsClassifier	79.78
			DecisionTreeClassifier	75.78
			SVC RBF kernel	74.87
			SVC	72.74
			AdaBoostClassifier	65.76
			QuadraticDiscriminantAnalysis	56.52
			GaussianNB	50.19
	Chroma + Melspectrogram + MFCC + contrast + tonnetz + ZCR, RSME	Yes	1D convolution (CNN)	97.09
			RandomForestClassifier	92.25
			KNeighborsClassifier	82.88
			SVC RBF kernel	79.46
			DecisionTreeClassifier	77.97
			SVC	73.64
			AdaBoostClassifier	68.60
			QuadraticDiscriminantAnalysis	58.91
			GaussianNB	52.00

**Table 4:** Recognition accuracy on individual emotion classes of BAVED.

Model	Low (%)	Medium (%)	High (%)
BAVED	97.45	95.87	97.76

**Table 5:** Recognition accuracy on individual emotion classes of ANAD.

Model	Angry (%)	Happy (%)	Surprised (%)
ANAD	98	91	96

**Table 6:** Recognition accuracy on individual emotion classes of SAVEE.

Model	Angry (%)	Disgust (%)	Fear (%)	Happy (%)	Neutral (%)	Sad (%)	Surprise (%)
SAVEE	78	75	79	83	99	71	87

**Table 7:** Recognition accuracy of 1D CNN on ANAD and SAVEE.

Model	Accuracy (%)	F1 score (%)	Recall (%)	Precision (%)
ANAD	96.44	95.46	95.17	95.82
SAVEE	83.33	83.579	83.579	83.579

**Table 8:** Summary of accuracies (%) obtained by various authors using BAVED database.

Method	Model	Accuracy (%)
[63]	wav2vec2.0	89
Ours	1D CNN	97.09

**Table 9:** Summary of accuracies (%) obtained by various authors using ANAD database.

Method	Model	Accuracy (%)
[64]	Linear SVM	96.02
Ours	1D CNN	96.44

**Table 10:** Summary of accuracies (%) obtained by various authors using SAVEE database.

Method	Model	Accuracy (%)
[65]	VACNN + BOVW	75
[66]	DCNN + CFS + SVM	82.10
Ours	1D CNN	83.33

## Data Availability

The data set is available at the following link: https://www.kaggle.com/a13x10/basic-arabic-vocal-emotions-dataset.
